# Use of Oral and Intravenous Tranexamic Acid in Total Knee Arthroplasty and Total Hip Arthroplasty Procedures

**DOI:** 10.7759/cureus.75741

**Published:** 2024-12-15

**Authors:** Taylor J Manes, Brendan E Kelly, Chris Main

**Affiliations:** 1 Orthopedic Surgery, OhioHealth Doctors Hospital, Columbus, USA; 2 College of Osteopathic Medicine, Des Moines University, Des Moines, USA; 3 General Orthopedics, Midwest Bone and Joint Center, Macon, USA

**Keywords:** drain output, hematocrit, postoperative blood loss, total knee arthroplasty (tka), tranexemic acid

## Abstract

Introduction: Tranexamic acid (TXA) is an antifibrinolytic drug commonly used in total knee arthroplasty (TKA). Intravenous (IV) and topical TXA therapy have been extensively studied and shown to reduce blood loss, length of hospital stay, and blood transfusion rates following TKA. Despite the extensive literature regarding IV and topical TXA in orthopedics, there is a current dearth of studies analyzing oral usage. The primary purpose of this randomized controlled study is to compare post-surgical blood loss with the use of IV and oral TXA following a TKA.

Methods: In this multicenter, prospective, controlled, randomized study, patients were randomized to receive 1.95 grams of oral TXA two hours preoperatively or 15 mg/kg (1 gram max) of IV TXA mixed in 100 mL of normal saline preoperatively. Intraoperatively, each patient received a combination of 500 mg TXA mixed with 25 mL of normal saline injected into the suction drain and clamped for 30 minutes postoperatively. The primary outcome was absolute (g/dL) change in hemoglobin levels at 24 hours postoperatively. Secondary outcomes included a percent change in hematocrit levels and total drain output at 24 hours postoperatively. Power analysis determined that 40 patients were required in each group.

Results: About 24 patients received IV TXA and 14 patients received oral TXA. The mean decrease in hemoglobin 24 hours postoperatively was not greater (p=0.12) with the use of oral TXA compared to IV TXA (1.70 g/dL vs. 2.50 g/dL, respectively). The mean decrease in hematocrit 24 hours postoperatively in the treatment (oral TXA) group was not greater (p=0.18) than the control (IV TXA) group (5.3% vs. 7.0%, respectively). Both changes in hemoglobin and hematocrit resulted in a normal distribution of data and satisfactory one-sided T-test values (p=0.98 and p=0.92, respectively). The mean drain output measured 24 hours postoperatively in the treatment group was significantly (p=0.04) less than the control group (47.5 mL vs. 170 mL, respectively). All tests performed were one-sided T-tests.

Discussion: Compared to IV TXA, we found oral administration to have no significant difference in hemoglobin (p=0.12) or hematocrit (p=0.18) loss. These findings may support the utilization of oral TXA as a valid alternative to the IV route. We found that patients who received oral TXA produced significantly lower drainage output when compared to IV (47.5 mL and 170 mL, respectively; p=0.04). Persistent wound drainage following TKA has been correlated with increased rates of periprosthetic joint infections, residual pain, and reoperation. Further work is needed to assess the effect of postoperative drainage quantity on the recovery of TKA.

Conclusion: Based upon the data gathered, oral TXA does not result in greater loss of hemoglobin or hematocrit within 24 hours following surgery. However, total drain output was significantly less in the oral group after 24 hours following a TKA. With more subjects, we would expect to see the mean values between groups move closer to each other and fit well with the current literature. This suggests that oral TXA is non-inferior to IV TXA when comparing these variables following TKA. Our current study has limitations, including a small sample size and no blinding. Therefore, this study provided a limited quality of evidence. Confirmation with a blinded, randomized controlled trial and meta-analysis is required to report a higher quality of evidence.

## Introduction

Total joint arthroplasty is considered a major surgical procedure and can result in significant blood loss. Average drops have been reported to range from 1-2 liters in total hip arthroplasty (THA) and up to 2 liters in total knee arthroplasty (TKA) [1]. At times, this deficit may even require a blood transfusion. In fact, it has been reported in the literature that up to 37% of patients undergoing a THA and 25% undergoing a TKA require a blood transfusion for postoperative blood loss [1]. Therefore, research to investigate ways to reduce patient morbidity and mortality from major surgical procedures is being extensively studied. One pharmacologic method that has been shown to reduce postoperative blood loss after joint replacement surgery is tranexamic acid (TXA) [1]. This is an antifibrinolytic drug commonly used in total hip and knee arthroplasty procedures today.

TXA was originally discovered in 1962 as a synthetic lysine derivative that forms a reversible interaction with plasminogen and plasmin [1]. This drug dates back as early as 1997 when one of the first randomized studies investigated TXA and the reduction of blood loss following joint replacement surgery [2]. In that study, 75 patients who underwent a primary TKA were randomized to intravenous (IV) TXA or normal saline. The TXA group demonstrated a significant reduction in blood loss and transfusions without an increase in thromboembolic events.

IV and topical TXA therapy has been extensively studied and shown to reduce blood loss following TKA procedures. Topical and IV forms of TXA have been studied more thoroughly than oral administration of TXA. Benefits from the utilization of TXA in TKA include a reduction in the length of hospital stay, blood transfusion rates, and hemoglobin loss. This is a significant benefit to patients because blood transfusions alone are associated with an increased risk of bacterial infections, an increased hospital stay, and, inevitably, an increased cost [3]. A study by Gausden et al. reported an increased cost of over $1,731 per admission in patients who received a blood transfusion [3].

Multiple studies have demonstrated that oral TXA is non-inferior to IV TXA in orthopedic procedures in regards to Hb drop, total blood loss, transfusion rate, drain blood loss, and length of stay [4-6]. Despite this evidence, large trials are lacking in the orthopedic community to help substantiate these findings. With an associated reduction in morbidity and mortality, TXA has gained more popularity recently. A meta-analysis by Farrow et al. reported a reduction in the need for blood transfusions by 46% in patients with hip fractures who received TXA intraoperatively [7].

Given the paucity of literature on the comparison of IV versus oral TXA administration, it was our aim with this single-blinded clinical study to add to the medical field’s current knowledge of how to most effectively administer TXA in total joint replacement procedures. This article was previously presented as a meeting abstract at the 2022 AOAO Fall Annual Scientific Meeting in Chicago, USA, in November 2022. The preliminary results of this study were presented, which demonstrated similar findings.

## Materials and methods

About 38 individuals undergoing elective total knee or hip replacement were enrolled in this prospective, multicenter, controlled, and randomized study. All 38 operations were performed by the same surgeon at three different rural hospitals. TKAs were performed with the standard medial parapatellar technique. This approach consists of a proximal dissection of the medial cuff of the quadriceps tendon as well as a distal subperiosteal dissection of the patellar tendon from the tibia. THAs were conducted by the standard posterolateral approach. Patients were offered inclusion in the study if they were above the age of 18 years with end-stage osteoarthritis and had exhausted conservative management. Exclusion criteria consisted of a history of severe ischemic heart disease (New York Heart Association III and IV), chronic renal failure, liver cirrhosis, bleeding disorders, or a current regimen of anticoagulation therapy. They were also excluded if they were pregnant or incarcerated and lacked documentation of preoperative hemoglobin (Hgb) within 30 days prior to their procedure or a documented postoperative Hgb within 24 hours.

A computer-generated random allocation sequence, determining either IV or oral cohort, was composed prior to patient recruitment and stored on a secure server. The patient cohort was then determined based on the order in which their procedure was performed in accordance with the random allocation sequence. Upon enrollment in the study, patients were educated on both cohorts and remained unaware of their cohort allocation until the morning of surgery. Prior to the procedure, baseline Hgb and hematocrit (Hct) values were collected for each patient. For patients receiving only oral TXA, a one-time dosage of 1.95 grams (a total of three tablets; 650 mg per tablet) was given two hours prior to the incision. Patients receiving only IV TXA were administered one dose of IV TXA (15 mg/kg or 1 gram max) mixed in 100 mL of normal saline 10 minutes prior to the first incision. At the end of each procedure, every patient in this study received a combination of 500 mg of TXA mixed with 25 mL of normal saline, which was injected into the suction drain and clamped for 30 minutes postoperatively.

Data collection included absolute (in grams/deciliter) and relative (%) changes in Hgb levels collected at 24 hours postoperatively, total blood loss during the procedure, drain output at 24 hours postoperatively, number of blood transfusions, and total length of hospital stay. Due to variable operative times, lab orders were specified and were subsequently collected at 24 hours postoperatively for each patient. All data was collected via electronic medical records at the hospital in which the procedure took place. Patient identifiers were removed, and data was subsequently stored on a single, secured external drive for statistical analysis. One-sided T-tests were performed for comparison of the two cohorts.

## Results

**Figure 1:**
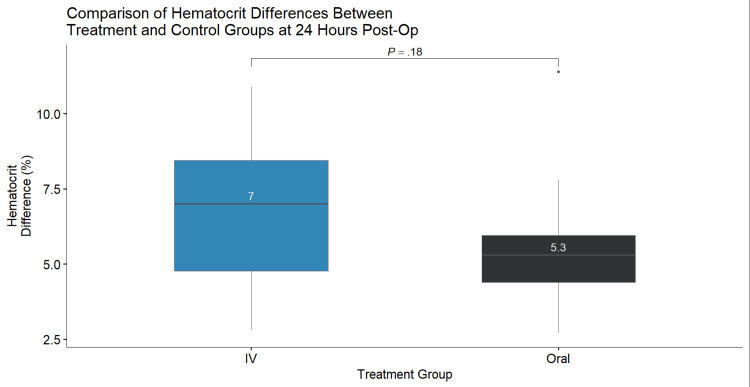
Comparison of hematocrit differences between treatment and control groups at 24 hours postoperatively.

**Figure 2:**
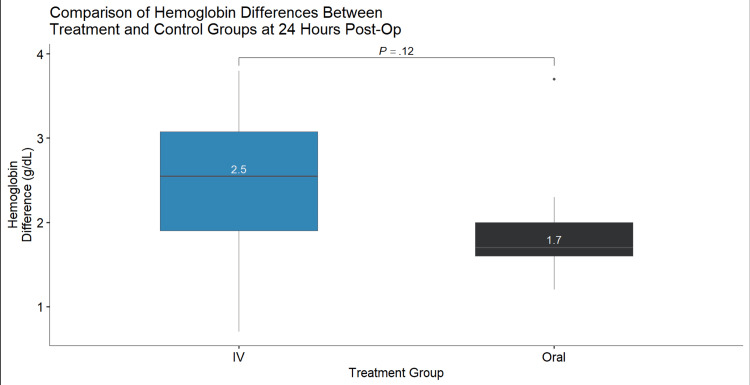
Comparison of hemoglobin differences between treatment and control groups at 24 hours postoperatively.

**Figure 3:**
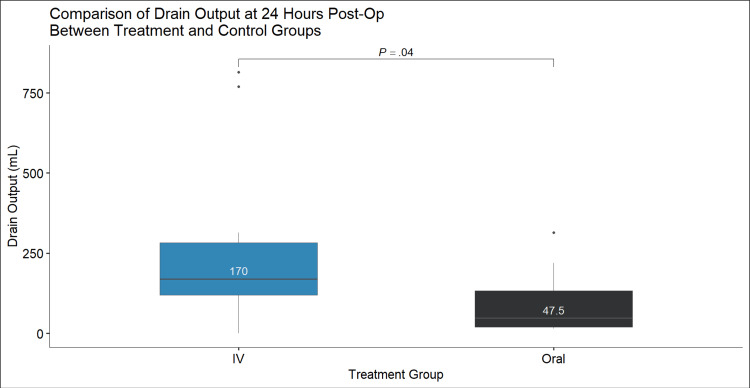
Comparison of drain output at 24 hours postoperatively between treatment and control groups.

About 24 patients received IV TXA and 14 patients received oral TXA. The mean decrease in hemoglobin 24 hours postoperatively was not greater (p=0.12) with the use of oral TXA compared to IV TXA (1.70 g/dL vs. 2.50 g/dL, respectively) (Figure 1). The mean decrease in hematocrit 24 hours postoperatively in the treatment (oral TXA) group was not greater (p=0.18) than the control (IV TXA) group (5.3% vs. 7.0%, respectively) (Figure 2). Both changes in hemoglobin and hematocrit resulted in a normal distribution of data and satisfactory one-sided T-test values (p=0.98 and p=0.92, respectively). The mean drain output measured 24 hours postoperatively in the treatment group was significantly (p=0.04) less than the control group (47.5 mL vs. 170 mL, respectively) (Figure 3). All tests performed were one-sided T-tests. There was no significant difference among collected demographics including age, weight, height, and BMI (p=0.61, p=0.43, p=0.27, p=0.837, respectively). Mean age was 67 and 69 years, mean weight was 97.79 and 104.08 kg, mean height was 1.66 and 1.70 m, and mean BMI was 35.5 and 36.2 kg/m^2^ in the IV and oral TXA cohorts, respectively. The oral TXA cohort consisted of eight females and six males, whereas the IV cohort consisted of 14 females and 10 males. Estimated blood loss between the two groups was found to be non-significant between the IV and oral groups at an average of 48 cc and 42 cc, respectively (p=0.64).

## Discussion

When compared to IV TXA, we found oral administration to have no significant difference in postoperative decrease in hemoglobin (p=0.12) or hematocrit (p=0.18). This is congruent with the literature comparing IV and oral TXA blood-sparing effects [1-3,8-10]. While similar in efficacy, the decision of TXA route administration is multifactorial, including the required cost and resources. As elaborated by prior investigators, oral TXA offers the benefit of not requiring additional IV access, specialized nursing care, or electronic drug-delivery systems [8]. If utilized in the approximate 700,000 primary TKA procedures performed annually within the United States, oral TXA could save our healthcare system $23 to $67 million dollars per year depending on the IV formulation chosen [9].

In addition, we found that patients who were administered oral TXA produced a significantly lower quantity of drainage when compared to IV at 24 hours postoperatively (47.5 mL and 170 mL, respectively; p=0.04). A study by Patel et al. found that higher drain output in both THA and TKA was associated with a longer time until the wound was dry [10]. Persistent wound drainage (PWD) following TKA has been correlated with multiple complications such as increased rates of periprosthetic joint infections, residual pain, and reoperation [11]. The International Consensus Meeting defined PWD to be greater than 2x2 cm of drainage beyond 72 hours postoperatively [11]. While this timeline was relatively aggressive when compared to the previous definitions of PWD, it was made in an attempt to minimize the need for invasive interventions. These guidelines, however, only offer benefits when the provider is knowledgeable of the signs associated with wound infection and maintains a high index of suspicion. Signs and symptoms of wound infection present both systemically and locally. Local signs consist of the persistence of a sinus tract, warmth, purulent discharge from the wound, and painful erythema. Systemic signs may present as fever, chills, or tachycardia [11].

Periprosthetic infections are one of the most common complications succeeding total joint arthroplasty. Recently, in a global meta-analysis, periprosthetic infection in non-diabetic patients occurred in 1.2% of cases compared to 1.9% in diabetic patients [12]. Superficial surgical site infections (SSSIs) leave patients susceptible to deep wound infections. A retrospective study by Saleh et al. assessing potential patient risk factors for SSSI found only postoperative hematoma formation and PWD to be significantly correlated with the formation of SSSI [13]. Patients with PWD lasting five days or more are 12.5 times more likely to develop infections [13]. After a review of 11,785 arthroplasties, Jaberi et al. recommend prompt surgical debridement in patients presenting with greater than seven days of PWD due to a high probability of infection [14]. The literature is lacking in depth and consistency with recommendations on how to optimize the timing of surgical intervention in PWD. More high-powered studies need to be conducted to present a high level of evidence regarding this.

The decision in regard to the timing of TXA administration was made to maintain congruence with prior literature [15]. Although the timing of oral versus IV differed by 2 hours and 10 minutes preoperatively, Pilbrant et al. expressed that timing had no significant effect on outcomes [16]. While current literature consistently describes the administration of oral TXA two hours preoperatively, the analysis of variable timing protocols is lacking. Further research is required to explore the optimal regimen.

Our current study has limitations, including a small sample size and no blinding. Data collection was discontinued prior to adequate power archival due to a lack of on-site researchers and an inability to collect data from remote locations. Additionally, data collection was not continued beyond 24 hours postoperatively. This reduced our ability to assess for delayed alterations in Hb, Hct, and drainage output. However, we randomized patients into each group via a computer-generated program. This assisted us in mitigating confirmation bias when selecting patients. This study was also performed at four different hospitals and by one surgeon. Reducing the number of surgeons performing the procedure helped us eliminate different surgical techniques that could have affected blood loss or drain output. Therefore, this study provided a limited quality of evidence. Confirmation with a double-blinded, randomized controlled trial and meta-analysis is required to report a higher quality of evidence.

## Conclusions

With more subjects, we would expect to see the mean values of postoperative blood loss between groups move closer to each other and fit well with the current literature. This suggests that oral TXA is non-inferior to IV TXA when comparing blood loss following a TKA. While larger studies are required to substantiate a widespread change to an oral TXA protocol, the decrease in postoperative drainage for the oral TXA group may suggest an avenue for a decreasing incidence of PWD. This benefit, in conjunction with the financial superiority of oral TXA, may support the utilization of oral TXA as a valid alternative to the more widely accepted IV formulation.
